# Supplementary figures and images for: A New Disease Caused by an Unidentified Etiological Agent Affects European Salamanders
Source: Animals (Basel). 2022 Mar 10;12(6):696. doi: 10.3390/ani12060696 (PMC8944795; doi:10.3390/ani12060696)

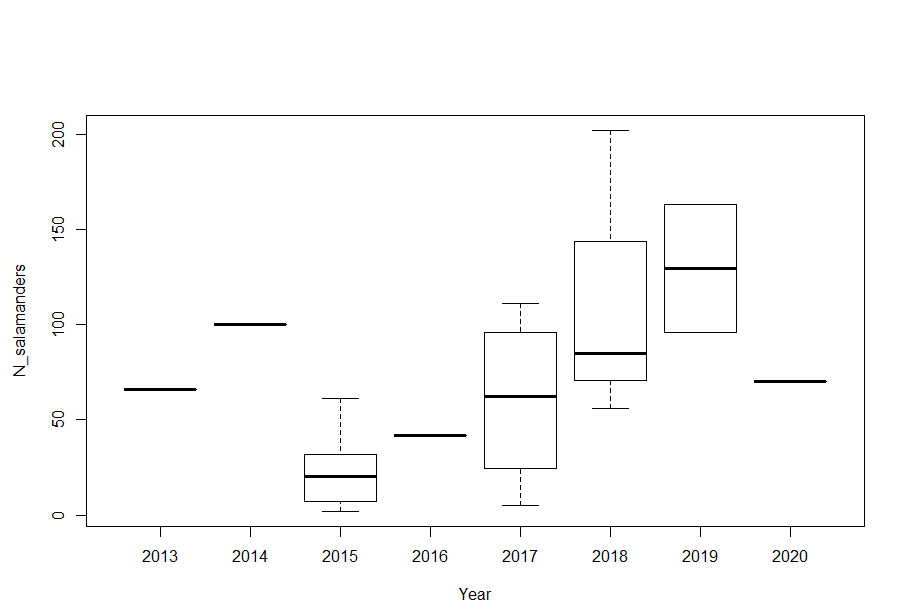

Supplement: Supplementary file 1 [file animals-12-00696-s001.zip › Figure S1.tiff]

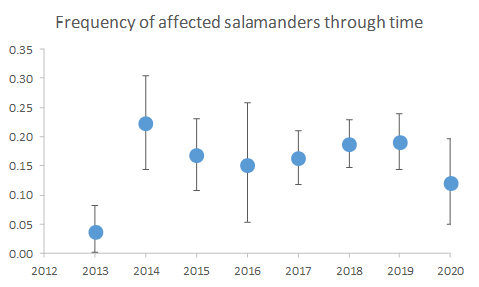

Supplement: Supplementary file 1 [file animals-12-00696-s001.zip › Figure S2.tif]
